# Study on Influence of Mechanical Behavior of AZ31 Magnesium Alloy Based on GTN Damage Modeling

**DOI:** 10.3390/ma18010090

**Published:** 2024-12-29

**Authors:** Peijie Wang, Chuanchuan Ma, Chun Xue, Zhibing Chu

**Affiliations:** School of Materials Science and Engineering, Taiyuan University of Science and Technology, Taiyuan 030024, China; m18935423607@163.com (P.W.); b202314110021@stu.tyust.edu.cn (C.M.); b202114310004@stu.tyust.edu.cn (C.X.)

**Keywords:** AZ31 magnesium alloy, GTN damage model, mechanical property, mechanism injury, parameter calibration and sensitivity analysis, micro–macro coupling analysis

## Abstract

Damage mechanisms are a key factor in materials science and are essential for understanding and predicting the behavior of materials under complex loading conditions. In this paper, the influence of different directions, different rates and different model parameters on the mechanical behavior of AZ31 magnesium alloy during the tensile process is investigated based on the secondary development of the VUMAT user subroutine based on the GTN damage model and verified by the tensile experiments at different loading rates and in different directions. The results show that AZ31 magnesium alloy exhibits significant differences in mechanical properties in radial and axial stretching, where the yield strength is lower in the radial direction than in the axial direction, and the elongation is the opposite. Moreover, the maximum stress and elongation of the material decreased with the increasing tensile rate, revealing the importance of the loading rate on the material properties. Compared with the existing studies, this paper determines the GTN model parameters of the AZ31 magnesium alloy extruded state bar by theresponse surface method combined with the optimization algorithm and obtains the parameter set that can accurately describe the damage behavior of this material. The study also found that the nucleation-averaged plastic strain ($\varepsilon_{N}$) has the most significant effect on the maximum stress and fracture point of the stress–strain curve by the sensitivity analysis of six key parameters of the GTN model, while the other parameters change the shape of the curve and the local features to different degrees. Further analysis shows that the differences in yield strength and elongation can be attributed to the differences in basal slip, twinning behavior and dynamic recrystallization in the microstructure, which provides an important guidance for the optimization of the microstructure of AZ31 magnesium alloy. This study not only reveals the influence law of loading conditions on the mechanical properties of AZ31 magnesium alloy but also provides a theoretical basis and reference for understanding the damage mechanism of magnesium alloy and optimizing its mechanical properties.

## 1. Introduction

Damage and fracture behavior of metals is an important topic in engineering, and magnesium alloys play a key role in a number of industrial fields such as automotive manufacturing, aerospace and electronics [1,2,3] due to their excellent strength-to-weight ratios, superior thermal conductivity and good corrosion resistance [4,5,6]. However, the failure of magnesium alloys [7,8], especially the fatigue and fracture behavior under high loads and complex environmental conditions, directly affects the safety and reliability of structures and has been a focus of research and application attention within the industry. Among them, the in-depth study of the fracture behavior of magnesium alloy under corrosion fatigue and internal pressure conditions is of great significance for the application of oil and gas transportation pipelines in deep sea and other extreme environments, which helps to ensure the stable operation of pipeline transportation systems. At the same time, optimizing the machining process and joining technology (e.g., riveting and welding) of magnesium alloy components to enhance their fracture toughness and fatigue life is critical to enhancing their performance under harsh operating conditions. In addition, the study of the fracture behavior of magnesium alloys in extreme temperatures and radiation environments, as well as the development of material treatment technologies with oxidation and fatigue resistance, is particularly important for enhancing the reliability of aerospace vehicle structures and the stability of nuclear industry facilities. In particular, the development of radiation-damage-resistant magnesium alloys and their composites, and the systematic study of post-irradiation fracture mechanisms and long-term service stability, will help to significantly improve the safety and reliability of these critical areas. Therefore, a systematic study of the damage mechanism of magnesium alloys in different directions and tensile rates is of great practical value for improving material properties and enhancing product safety.

In the field of the damage failure of metallic materials, different metals show diverse failure modes due to their unique physical and chemical properties; not only magnesium alloys but also other metals such as high-strength steels and aluminum alloys have numerous differences in terms of damage failure. For example, Zhonghao Cui [9] and others calibrated the parameters of the damage model for X80 steel pipeline circumferential welds and its prediction of crack expansion behavior under simulated real working conditions and showed that the description of weld damage evolution can be significantly improved by the accurate calibration of parameters, thus optimizing the safety and stability of pipeline systems. Ouyang Xiao [10] et al. investigated the failure behavior of AA5052-H32 aluminum alloy die-less punched riveted joints under cross tensile and tensile shear loading conditions by experimental and simulation methods, analyzed the deformation, damage and destruction process of the joints under the two loading conditions, and conducted macro/micro fracture analysis to further reveal the failure behavior for the practical application of die-less punched riveted joints. Macroscopic/microcosmic fracture analysis is also conducted to further reveal the failure behavior for the practical application of moldless riveted joints.

Most of the current research on magnesium alloys focuses on the processing and performance enhancement of plates [11]. Through different processes, researchers have been able to significantly improve the microstructure of magnesium alloy plates, thereby enhancing tensile strength and ductility [12,13]. Compared to plates, magnesium alloy rods [14] are equally important in engineering applications. Rods are often used for components that carry large axial loads, which require extremely high-strength and high-fatigue properties. In order to gain a deeper understanding of the damage mechanisms in magnesium alloys, the GTN (Gurson–Tvergaard–Needleman) damage model was introduced as an analytical tool [15]. The GTN model is able to take into account the material porosity and nucleation behavior, thus effectively simulating the damage process of the material under different loading conditions. This model provides a new perspective to study the mechanical properties of magnesium alloys under complex loads and multidirectional stresses and helps to improve the understanding of their damage evolution characteristics. However, magnesium alloy sheets are usually in a rolled state, and their microstructure exhibits strong texture characteristics, with grains arranged along the rolling direction and showing obvious anisotropy. Magnesium alloy bars are mostly in an extruded state, and the grains undergo dynamic recrystallization during the extrusion process to form a more uniform and finer microstructure. This difference in process significantly affects the pore nucleation, growth and polymerization behavior of the material, resulting in large differences in GTN model parameters between plates and rods. Therefore, this paper aims at the microscopic characteristics of AZ31 magnesium alloy rods, combines simulation and optimization methods and recalibrates its GTN model parameters to improve the model’s prediction accuracy for the damage evolution of the rods under complex loading conditions.

Ji Hongchao [16] investigated the damage evolution of 7075 aluminum alloy under high temperature conditions by means of a finite element inverse calibration method based on the GTN model and concluded that the temperature has a significant effect on the void volume fraction and damage parameters of the material, which verified the accuracy of the method. Chen Xin [17] and other scholars studied the development and application of the GTN microscopic damage model, analyzed the typical correction methods of the GTN model and parameter calibration techniques and looked forward to the development trend of the model, which provides a reference for the research and application of the GTN model in the mechanical analysis of microscopic damage. By combining experimental observation and numerical simulation, Rui-ze Wang [18] successfully simulated the fracture initiation of AZ31 magnesium alloy sheets during hot stamping by applying the extended GTN damage model and Hill’s quadratic anisotropic yield criterion to the VUMAT subroutine and verified the accuracy of the model with experimental data. P.J. Zhao [19] used a modified GTN damage model, incorporating the Yld2000 anisotropic yield criterion, to investigate the damage and failure behavior of AZ31 magnesium alloy during warm stamping and embedded the material intrinsic equations into the VUMAT subroutine, which was applied to the formability analysis of a cellular phone casing. Addisu Negash Ali and other scholars [20] derived the optimized damage parameters for the plastic fracture behavior of AZ61 magnesium alloy deformed by Equal Channel Angular Pressing (ECAP) by means of response surface methodology (RSM) and GTN damage modeling as well as finite element simulation (FEM). Tairui Zhang and Yafan Zhao [21] investigated the parameter identification of the GTN model through experiments and finite element simulations for two ductile metals (SA516 and S30408), provided a systematic approach to calibrate all the eight parameters in the GTN model and verified the applicability of these parameters in predicting the failure behaviors of cracked and uncracked bodies. Through the improved GTN damage model, combined with the experimental data and finite element simulation of AZ31B magnesium alloy, Chen-Chen Zhao [22] investigated the damage evolution and crack extension mechanism in the upsetting and forming process and verified the validity of the model for the prediction of low-stress triaxial degree damage. H. Gholipour [23] used the GTN model and response surface method to calibrate the damage parameters of SAE 1010 carbon steel, combined with tensile experiments and numerical simulation, to analyze the toughness fracture behavior under different stress states and concluded that the improved GTN model can better predict the fracture location and crack extension direction. Yue Yin [24] used the GTN model and mesh sensitivity analysis to calibrate and simulate Q355D steel plates based on tensile experiments to study the net cross-section fracture behavior of steel plates containing different shapes of holes, verified the reliability of the GTN model in predicting the fracture loads and displacements and concluded that the mesh sizes have a smaller effect on the fracture prediction. These studies have demonstrated the value of the GTN model and its improved versions for understanding and predicting the damage and fracture behavior of materials in practical applications.

This paper is organized as follows: First, the importance of damage mechanism in materials science is introduced, and the GTN model is introduced. The second part details the theoretical development of the GTN model and the logical structure of the VUMAT user subroutine flow. The third part mainly describes the preliminary preparation of experimental materials and the setting of the simulation part. The fourth part shows the stress–strain curves obtained from experiments and simulations and compares and analyzes the effects of different orientations and different stretching rates on the mechanical behavior of the material. In addition, the influence of various parameters in the GTN model on the stress–strain curve is also deeply discussed, and the damage and fracture process of the material is simulated through simulation. Finally, the above analysis is summarized, and the conclusion is drawn.

## 2. GTN Damage Theory Model

**Figure 1:**
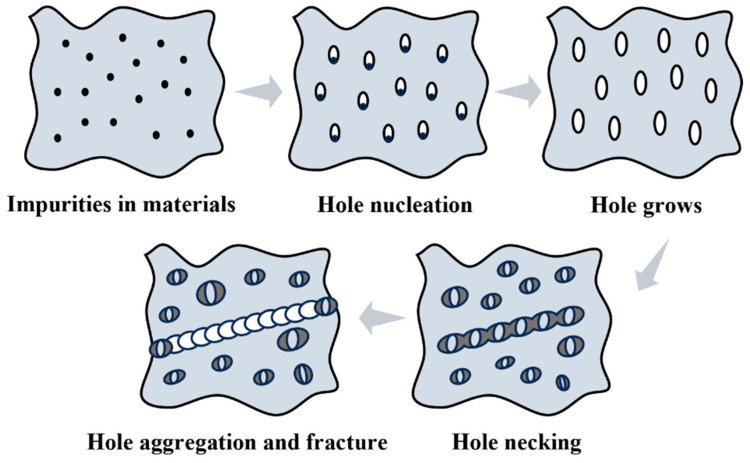
Schematic diagram of void expansion.

Metallic materials undergoing plastic deformation are usually accompanied by the evolution of microstructural damage and defects, such as cracks and voids, etc. Damage theory models [25,26] are developed to describe and predict these damage behaviors by mathematical means. Damage models can be broadly categorized into two types: continuous damage mechanics models and fine-scale damage mechanics models. The continuous damage mechanics model describes the damage process of the material on the macroscopic scale by introducing state variables without focusing on the microstructural changes of the material; on the contrary, the fine damage mechanics model focuses on the generation and evolution process of the microscopic damage inside the material from the microscopic scale as shown in Figure 1. The establishment of these models provides a theoretical basis for predicting the damage behavior of materials under different processing conditions. In the forming process of metallic materials, the generation and extension of cracks can be effectively predicted by means of finite element simulation and other means, thus helping to optimize the processing of the materials and prolong their service life.

The GTN model is an improvement of the Gurson model [27] proposed by Gurson in 1977, which aims at describing the evolution of voids in porous metallic materials during plastic deformation and its effect on the mechanical behavior of the material. The Gurson model combines the internal microscopic damage (e.g., voids) of the material with the macroscopic mechanical response by introducing the variable of porosity, which for the first time relates the void volume fraction to the yield criterion of the material, and Equation (1) is the Gurson model expression.

(1) $$\phi =\frac{q^{2}}{\sigma_{m}^{2}}+2f\cosh\left(\frac{3\sigma_{h}}{2\sigma_{m}}\right)-(1+f^{2})=0$$

When f=0, it represents no damage to the material, and the equation degenerates into the Von Mises yield criterion; when f=1, it represents a complete loss of loading capacity, i.e., total failure. The model assumes that the voids inside the material expand under external loads, resulting in a decrease in the yield stress of the material. When the porosity of the material increases to a certain level, the bearing capacity of the material gradually decreases, eventually leading to fracture. However, the Gurson model does not take into account the interaction between voids and the accelerating effect of void polymerization on the material failure.

To address these issues, Tvergaard and Needleman modified [28] the original Gurson model by introducing three correction parameters, $q_{1},q_{2}$ and $q_{3}$, to more accurately reflect the effect of void polymerization on material failure. The optimized posterior GTN yield function is as follows:

(2) $$\phi =\frac{q^{2}}{\sigma_{m}^{2}}+2q_{1}f^{*}\cosh\left(\frac{-3q_{2}\sigma_{h}}{2\sigma_{m}}\right)-(1+q_{3}f^{*2})=0$$

where q is Von Mises equivalent stress, $\sigma_{m}$ is the yield stress of the material and $q_{1},q_{2}$ and $q_{3}$ are three damage parameters, representing the interaction between voids. When $q_{1}=q_{2}=q_{3}=1$, Equation (2) degenerates to the original Gurson model. By Tvergaard and Needleman, through theoretical analysis and experimental studies, $q_{1}=1.5$, $q_{2}=1.5$ and $q_{3}={q_{1}}^{2}=2.25$ can be applied to most metals.

$\sigma_{h}$ is the hydrostatic or average stress of the material, which can be expressed as follows:

(3) $$\sigma_{h}=\frac{1}{3}tr(\sigma )=\frac{\sigma_{xx}+\sigma_{yy}+\sigma_{zz}}{3}$$

$tr\left(\sigma \right)$ is the trace of the stress tensor, and $\sigma_{xx},\sigma_{yy}\;\mathrm{and}\;\sigma_{zz}$ are the diagonal elements of the stress tensor, corresponding to positive stresses in the x,y and z directions, respectively.

$f^{*}$ is the material porosity, i.e., the volume fraction of voids within the material, as a function of the porosity f, as shown in Equation (4):

(4) $$f^{*}=\left\{\begin{array}{ll} f, & \;f\leq f_{c}\\ f_{c}+k(f-f_{c}), & \;f>f_{c} \end{array}\right.$$

(5) $$k=\frac{f_{u}^{*}-f_{c}}{f_{f}-f_{c}}$$

where $f_{c}$ is the critical void volume fraction of void polymerization, k is the void growth acceleration factor, $f_{f}$ is the void volume fraction of the material at fracture and $f_{u}^{*}$ is the porosity value when the stress is zero. When $f^{*}=0$, it indicates that there is no damage within the material and the yield function degenerates to the Mises yield function, which in the initial state is $f^{*}=f$.

For ductile metallic materials, the damage is considered isotropic in the GTN damage model because the damage-induced material anisotropy is usually insignificant. The total damage increment can be expressed as a change in void volume fraction, which consists of two components: growth of the original voids and nucleation of new voids. The increment of porosity can be expressed as follows:

(6) $$df=df_{\mathrm{grow}}+df_{\mathrm{nucl}}$$

where $df_{grow}$ is the growing portion of the original voids, and $df_{nucl}$ is the nucleated portion of the new voids. Since the matrix material is incompressible, according to the law of the conservation of mass, the growth of the original voids $df_{grow}$ depends on the macroscopic plastic volume strain.

(7) $$df_{\mathrm{grow}}=(1-f)d\varepsilon^{p}:I$$

where I is the second-order unit tensor.

The change in damage caused by the nucleation of new voids can be expressed as follows:

(8) $$df_{\mathrm{nucle}}=M_{1}d\sigma_{m}=Ad{\bar{\varepsilon }}_{m}^{p}$$

(9) $$A=\frac{f_{n}}{s_{N}\sqrt{2\pi }}=\exp\left(-\frac{1}{2}{\left(\frac{\varepsilon_{p}-\varepsilon_{N}}{s_{N}}\right)}^{2}\right)$$

$f_{n}$ is the volume fraction of voids in the two-phase material;

$\varepsilon_{p}$ is the macroscopic equivalent plastic strain;

$\varepsilon_{N}$ is the average plastic strain of nucleation;

$s_{N}$ is the standard deviation, indicating the degree of dispersion of the nuclearized strain.

This formulation describes the nucleation of new voids in the material and reflects the strain dependence of void nucleation through a statistical distribution. Where $\varepsilon_{N}=0.3$, $s_{N}=0.1$ are values obtained by Chu C and Needleman A [29] through extensive experimental studies applicable to most metallic materials.

Nahshon et al. [30] proposed a modification of the GTN model that takes into account shear damage by introducing a shear damage term specifically for the change in void volume fraction. The model corrects the GTN model by adding the effect of shear strain on the void volume fraction. Specifically, the shear damage correction term is expressed as follows:

(10) $$w(\sigma_{ij})=1-{\left(\frac{27J_{3}}{2\sigma_{eq}^{2}}\right)}^{2}$$

$J_{3}$ is the third invariant of the bias stress tensor to characterize the strength of the shear stress state, and $\sigma_{eq}$ is the equivalent stress.

**Figure 2:**
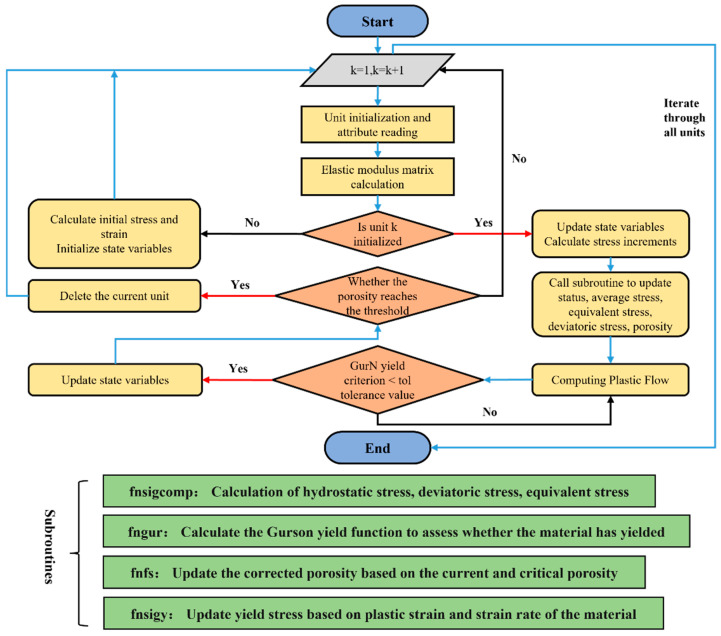
Schematic diagram of VUMAT subroutine flow.

Combined with the VUMAT subroutine, it is able to accurately simulate the stress–strain curve of the material, and by adjusting the parameters of void growth, aggregation, etc., the stress–strain data obtained from the simulation can be compared with that of the experiment. The following is the flowchart of the VUMAT subroutine as shown in Figure 2.

## 3. Experimental Design and Simulation Setup

### 3.1. Experimental Design

The experimental material used in this paper is the AZ31 magnesium alloy bar obtained by the extrusion process, and its main composition is shown in Table 1.

**Figure 3:**
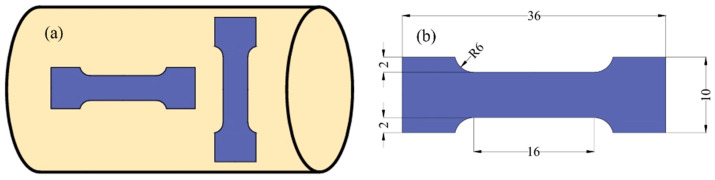
Schematic diagram of materials and specimens: (**a**) specimen orientation; (**b**) specimen dimensions.

Figure 3 illustrates the specimen orientation and dimensions of an AZ31 magnesium alloy bar. Figure 3a illustrates the specimen orientation, where the axial and radial directions represent cutting the specimen from the magnesium alloy bar in the direction of its axis and perpendicular to the axis, respectively. This difference in orientation is intended to investigate the anisotropy of materials in different crystallographic orientations, i.e., there may be significant differences in the mechanical properties of materials in different directions.

Figure 3b, on the other hand, provides the specific dimensions of the specimens, which were determined according to the national standard GB/T228.1-2021 [31] to ensure the standardization of the specimens and the comparability of the experimental results. The thickness of the specimens was standardized to 2 mm, which was to obtain a consistent stress–strain response in the experiments. The geometry and dimensions of the specimen are critical to the accuracy of the experiment as they directly affect the stress distribution and possible deformation patterns.

In the experimental process, the stress and strain of the specimen in the tensile process were measured in real time using a high-precision force transducer to ensure the accuracy of data collection. The temperature of the experimental environment was maintained at about 20 °C to keep stable experimental conditions to reduce the interference of environmental factors on the experimental results. In the axial direction, a total of three different tensile rates of 0.1 mm/min, 0.5 mm/min and 1 mm/min were used in the experiments to investigate the effect of the strain rate on the mechanical properties of the materials through the change of the tensile rate.

### 3.2. Simulation Setup

The model is created by importing or drawing the geometry of the tensile specimen and then setting the desired material parameters, including density, elasticity and plasticity data and GTN-model-related parameters. The GTN model parameters determine the accuracy of the description of the material damage behavior, and the setting of the GTN model parameters is particularly important to ensure the accuracy of the simulation results. Tvergaard and Chu have already provided values for $q_{1},q_{2},q_{3},\varepsilon_{N}\;\mathrm{and}\;{\;s}_{N}$ through extensive experiments, respectively, and the remaining ${f_{0},f}_{n},f_{c}\;\mathrm{and}\;f_{f}$ parameters can be determined by a variety of methods. Among them, the microscopic observation method [32] is used to derive the parameters related to the void volume fraction by further processing the derived results through metallurgical microscopy or scanning electron microscopy (SEM). However, due to the inhomogeneity of the distribution of internal voids in the material during the processing stage, the parameters derived from the micro-observation method can only be limited to the observation area; therefore, their accuracy is limited and can only be used as an auxiliary means to provide a reasonable range of parameters. Another method is the representative volume unit method (RVE method) [33], which describes the relationship between the macroscopic mechanical behavior of a material and its microstructure through the use of a representative volume unit and determines the damage parameters of a material by applying material properties to a single unit and loading it to simulate the damage and failure process of the material. The most commonly used method is the finite element inversion method [34,35], which performs the simulation by initially calibrating the model parameters and combining them with an optimization algorithm to select different GTN model parameters. The simulation results are compared with the experimental data to gradually narrow the gap between the two, so as to obtain more accurate GTN model parameters. The parameters of the GTN model in this paper are determined by combining the finite element inversion method, the response surface algorithm and the genetic algorithm, as shown in Table 2 below.

After completing the material parameterization, the appropriate loads and boundary conditions were applied according to the experimental program. In the simulation process, in order to ensure the accuracy and stability of the simulation, we adopt Dynamic Explicit, which is particularly suitable for the case of high nonlinearity, large deformation and material damage and can effectively deal with sudden failure phenomena and reduce the convergence problem in the simulation process. The VUMAT subroutine, which also corresponds to the display analysis, is able to better handle complex material behavior and damage evolution processes, making the simulation results more accurate and reliable.

**Figure 4:**
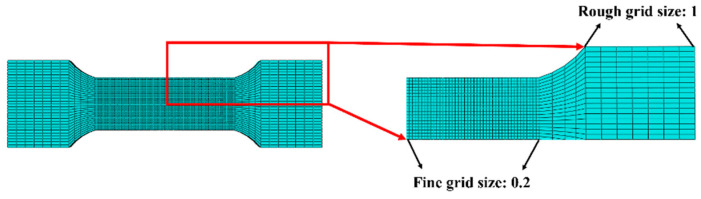
Schematic diagram of grid division.

For meshing, we used C3D8R grid cells (8-node reduced integral 3D solid cells). This cell type has the advantages of high computational efficiency and being able to deal with large deformation problems effectively, ensuring a balance between simulation accuracy and computational efficiency. The reduced integration method of the C3D8R cell can reduce the calculation time while ensuring the calculation accuracy, which is especially suitable for large-scale simulation problems in dynamic explicit analysis, and the mesh delineation of the AZ31 magnesium alloy specimen is shown schematically in Figure 4. As can be seen from the figure, the mesh in the middle region is obviously more detailed than the two end sections, and this design ensures that more accurate calculation results can be obtained in the critical stress region, thus improving the overall reliability of the simulation analysis.

## 4. Analysis of Experimental Data and Simulation Results

### 4.1. Comparative Analysis of Tensile Fracture Behavior and Fracture Profile

**Figure 5:**
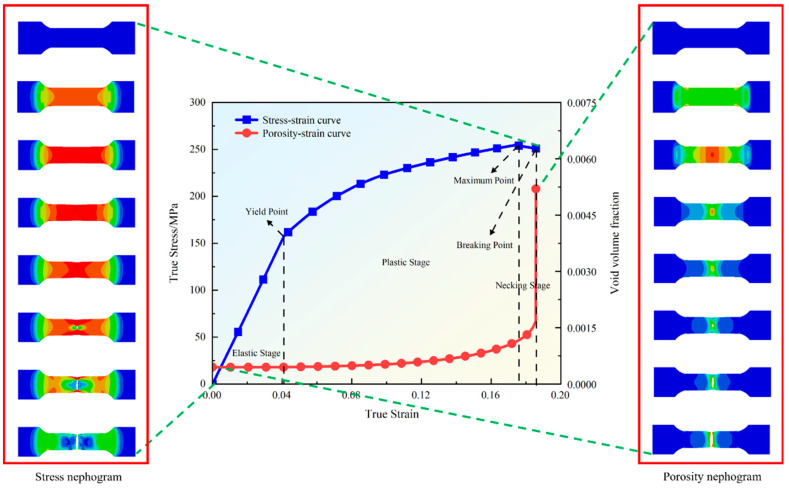
Stress–strain and porosity–strain curves and corresponding stress and porosity nephograms.

Uniaxial tensile simulation is used to study the mechanical behavior and void evolution of the material during the tensile process. As Figure 5 shows the stress–strain curve and porosity–strain curve during the simulation, the stress nephograms and porosity nephograms at different strains with more obvious changes in the tensile process are given on the left and right, respectively.

The stress–strain curve demonstrates the typical pattern of stress variation with strain during the stretching of a material. In the elastic phase, the stress and strain of the material show a linear relationship, and, after reaching the yield point, the curve enters the plastic phase, showing a flat tendency and permanent deformation of the material. After reaching the ultimate load point, it enters a small part of the gentle decline stage; the material from the uniform deformation transition to the local deformation, manifested as a concentrated necking, although the stress is decreasing, but the strain is still increasing until the necking region cannot withstand further deformation, reaches the fracture point and eventually fractures. The stress nephograms under different strains can be seen from the initial elastic stage, the stress is relatively uniformly distributed in the specimen, with the increase in displacement, the stress is gradually concentrated in the middle necking region of the specimen and, finally, when the material is fractured, the stress at the fracture reaches the maximum value, which shows the destructive characteristics of the stress concentration region.

The porosity–strain curve, on the other hand, demonstrates the variation of microscopic voids within the material with strain. At the beginning of the damage, the change in porosity is insignificant and relatively flat, and as the strain increases, the porosity increases dramatically near the fracture. This phenomenon indicates that the microscopic voids within the material do not initially grow significantly during the tensile process, and it is not until the plastic deformation is concentrated that the voids begin to expand and coalesce dramatically and ultimately lead to the formation of macroscopic cracks and the failure of the material at fracture. The porosity nephograms at different strains are similar to the stress nephograms in that the change in porosity is not obvious in the elastic phase, but, with tensile deformation, the porosity gradually increases and concentrates in the region of material necking. At fracture, porosity increases dramatically from indication to interior, forming a macroscopic crack through the specimen, corresponding to mechanical damage.

**Figure 6:**
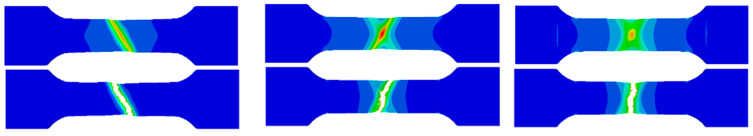
Simulation of tensile specimen fracture critical point and different fracture pattern nephograms.

**Figure 7:**
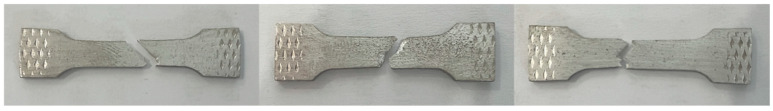
Experimental tensile specimens with different fracture profiles.

Figure 6 shows the cloud view of porosity distribution of AZ31 magnesium alloy tensile specimens at the fracture critical point as well as different fracture morphologies obtained by simulation; the change in color indicates the stress concentration region. Figure 7 shows the different fracture morphologies of AZ31 magnesium alloy specimens after the actual tensile experiments, three different fracture patterns are shown from left to right, and the common feature of these fractures is that all of them show the fracture behavior of the material during the tensile process, which forms a fracture pattern with the expansion of the necking in the middle. A high degree of agreement was achieved between the experimental results and the simulation predictions in terms of the morphology and location of the fracture surfaces, which verifies the accuracy of the GTN model in predicting the tensile behavior of AZ31 magnesium alloy materials, confirms the validity of the simulation methodology and provides a reliable tool for the study of material fracture, which can help to gain an in-depth understanding of the damage mechanism of the material and optimize its mechanical properties.

### 4.2. Effect of Different Orientations of Stretching on Stress–Strain Curves

**Figure 8:**
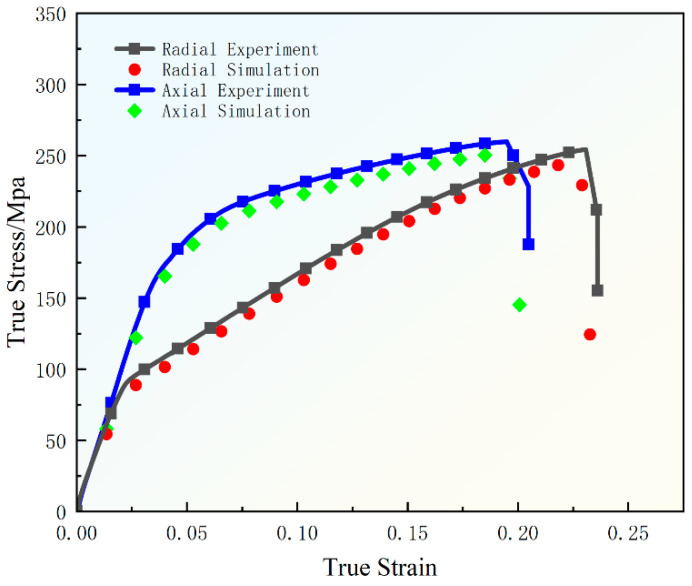
Axial and radial experimental and simulated stress–strain curves.

The stress–strain behavior of AZ31 magnesium alloy rods in different directions was explored through the experiments of the tensile rate of 1 mm/min in axial and radial directions and the corresponding finite element simulation simulations combined with the GTN damage model. As shown in Figure 8, the experimental data and simulation results in axial and radial directions are in good agreement, especially at the maximum stress point and the fracture stress point, and the simulation results can predict the trend of the experimental data well. This result indicates that the simulation model used possesses a high degree of accuracy.

A comparative analysis reveals that there is a significant difference between axial and radial directions in terms of yield stress and maximum strain values. The elastic phase is shorter in the radial direction, and its yield stress is significantly lower than that in the axial direction, which indicates that in the radial direction the material enters the plastic deformation phase earlier. At the same time, the maximum strain values in the radial direction are higher than in the axial direction, indicating that the material has better ductility in the radial direction. On the contrary, although the axial direction exhibits higher yield stresses and longer elastic deformation phases, the maximum strain values are smaller, indicating relatively poor ductility, reflecting the apparent anisotropy of the material in different directions. In addition, the axial and radial specimens did not show a significant difference at the maximum stress point and the fracture stress point, which indicates that the fracture behavior of AZ31 magnesium alloy rods is similar after reaching the maximum load-carrying capacity, regardless of the direction. Overall, there are differences in yield strength and elongation in different directions, and yield strength and elongation show a certain inverse relationship. This is mainly due to the unique hexagonal close-packed (HCP) crystal structure of magnesium alloys, whose yield strength and elongation are significantly affected by grain weave, orientation and microscopic deformation mechanisms. Specifically, the difference in yield strength may be related to the ease of the activation of slip systems (e.g., basal and prismatic slip) and twinning behavior due to differences in grain orientation, while the variation in elongation is closely related to dislocation density, grain boundary slip and twinning contributions. In addition, the distribution of precipitated phases and dynamic recrystallization behavior in the microstructure may also exhibit significant differences in different directions, further affecting the yield strength and elongation. For example, when the grain orientation is aligned with the loading direction, basal slip is more likely to occur, reducing the yield strength, while perpendicular to the loading direction, the slip system is limited and the yield strength increases. At the same time, changes in the degree of dynamic recrystallization may alter the grain size distribution and dislocation density, which may have an effect on the elongation. These characteristics indicate that the microstructure of AZ31 magnesium alloy determines its mechanical properties to a large extent, and this anisotropy is closely related to the intrinsic characteristics of the crystal structure of the magnesium alloy, which is of great significance for the prediction and application of its mechanical behavior. Optimizing the microstructure for the specific conditions of the application not only significantly improves the properties of the material but also plays a decisive role in ensuring the reliability and stability of the material in a particular application.

### 4.3. Effect of Different Tensile Rates on Stress–Strain Curves

By changing the axial stretching speed, a series of tensile specimen experiments were carried out on AZ31 magnesium alloy bar in the axial direction using 1 mm/min, 0.5 mm/min and 0.1 mm/min, respectively, with the purpose of investigating the effect of different stretching rates on the mechanical behavior of the material, and corresponding simulation simulations were carried out, which illustrated that the simulation model can capture the mechanical response of the material in different loading rates.

**Figure 9:**
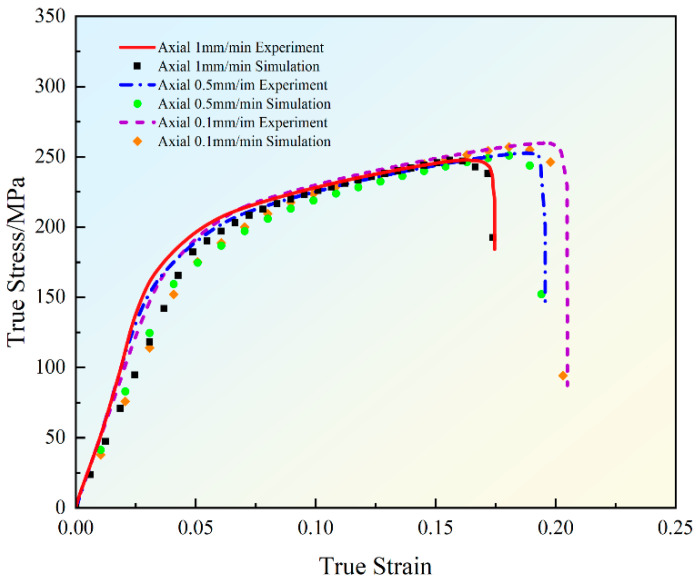
Experimental and simulated stress–strain curves for different rates in axial direction.

The experimental and simulated stress–strain curves at different rates in the axial direction are shown in Figure 9. Through the careful analysis of the experimental data, it is found that although the yield stress and tensile strength of AZ31 magnesium alloy increase slightly with the increase in the tensile rate, the overall change is not significant, which shows that the alloy is weakly sensitive to the tensile rate under room temperature conditions. However, the stress–strain curves at different tensile rates show significant differences during the plastic deformation stage, especially when approaching the fracture point. At a tensile rate of 1 mm/min, the maximum point stress is about 247.8 MPa, and the strain is about 16.3%; additionally, the fracture point stress is about 217.9 MPa, and the strain is about 17.5%. At a tensile rate of 0.5 mm/min, the maximum point stress is about 252.7 MPa, and the strain is about 18.8%; additionally, the fracture point stress is about 222.1 MPa, and the strain is about 19.6%. At a tensile rate of 0.1 mm/min, the maximum point stress is about 259.9 MPa, and the strain is about 19.5%; additionally, the fracture point stress is about 229.6 MPa, and the strain is about 20.5%. It is found that the tensile rate is negatively correlated with the stress and strain values at the maximum point, and the stress and strain values at the fracture point; the smaller the tensile rate, the larger the stress and strain values at the maximum point and the fracture point. The reason for this phenomenon is as follows: lower tensile rates lead to a more pronounced strain hardening phenomenon, which is facilitated by more adequate dislocation motion and dynamic recovery processes within the material at lower rates. In addition, the maximum strain values of the specimens before fracture at different tensile rates are different, indicating the effect of tensile rate on the plastic deformation capacity of the material, and lower tensile rates help to increase the plastic deformation capacity of the material, which allows the material to withstand greater strains before fracture.

### 4.4. Effect of Change of GTN Model Parameters on Stress–Strain Curves

**Figure 10:**
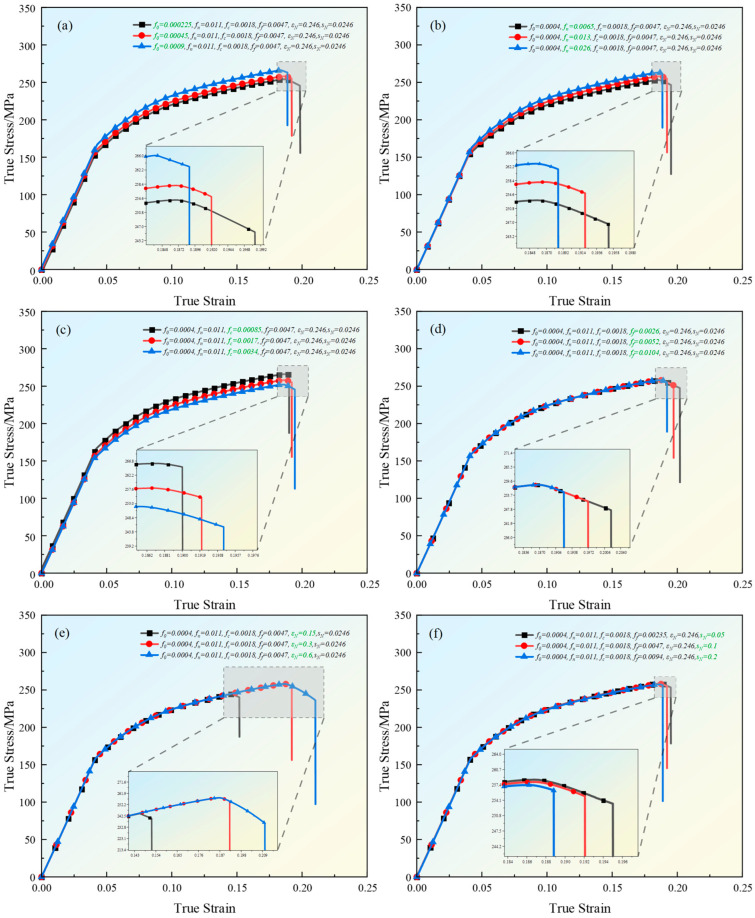
Simulation stress–strain curves under various parameters: (**a**) the influence of $f_{0}$ on the simulation curve; (**b**) the influence of $f_{n}$ on the simulation curve; (**c**) the influence of $f_{c}$ on the simulation curve; (**d**) the influence of $f_{f}$ on the simulation curve; (**e**) the influence of $\varepsilon_{N}$ on the simulation curve; (**f**) the influence of $s_{N}$ on the simulation curve.

An in-depth analysis of the effects of six key parameters in the GTN model on the stress–strain behavior of the material is carried out by using simulation combined with the single variable method. As shown in Figure 10 below, this series of plots shows the simulation results obtained by tuning the six parameters $f_{0},f_{n},f_{c},f_{f},\varepsilon_{N}\;and\;s_{N}$ in the GTN model, respectively, and each subplot (a), (b), (c), (d), (e) and (f) corresponds to the change of one parameter, showing the curves obtained when scaling up and down by a factor of two from the original values.

Figure 10a shows the effect of changing $f_{0}$ on the stress–strain curve. By comparing the curves under different $f_{0}$ values, it is analyzed that as $f_{0}$ gradually increases, the stress of the entire curve after the yield point also gradually increases. The maximum point stress increases from 254.1 MPa when $f_{0}$ = 0.000225 to 266.1 MPa when $f_{0}$ = 0.0009, and the fracture point stress increases from 245.5 MPa when $f_{0}$ = 0.000225 to 263.1 MPa when $f_{0}$ = 0.0009, but the strain at the fracture point decreases from 19.8% to 18.9%. The change of $f_{0}$ has little effect on the yield point. Regardless of the initial porosity, the yield stress of the material is close to 155.03 MPa. It shows that with the increase in $f_{0}$, the maximum load-bearing capacity and fracture limit of the material also increase, but the elongation decreases slightly. When $f_{0}$ is larger, there are more voids initially, and, due to the early growth and consolidation of the voids, the stresses within the material are redistributed so that the stresses in the undamaged region are concentrated, leading to higher stress levels in the localized region, which results in higher values of stress at the point of maximum stress and at the point of rupture, and the early growth of the voids results in earlier void polymerization and rupture, which results in a decrease in the strain at rupture.

Figure 10b shows the effect of changing $f_{n}$ on the stress–strain curve. By comparing the curves under different $f_{n}$ values, it is analyzed that as $f_{n}$ gradually increases, the stress of the entire curve gradually increases in the plastic stage, among which the maximum point stress increases from 253.03 MPa when $f_{n}$ = 0.0065 to 263.01 MPa when $f_{n}$ = 0.026, and the fracture point stress increases from 246.5 MPa when $f_{n}$ = 0.0065 to 261.5 MPa when $f_{n}$ = 0.026, but the strain at the fracture point decreases from 19.5% to 18.9%. It shows that $f_{n}$ has the same influence on the maximum load-bearing capacity, fracture limit and elongation as $f_{0}$. The increase in $f_{n}$ accelerates the growth rate of voids, making the material show stronger hardening ability on a macro scale. At this stage, the material continuously absorbs energy and disperses stress through plastic deformation, thus improving the maximum bearing capacity and ultimate fracture strength. However, due to the rapid growth of voids, microcracks inside the material are formed earlier and expand faster, and local stress concentration becomes more serious, resulting in faster fracture and a slight decrease in elongation.

Figure 10c shows the effect of changing $f_{c}$ on the stress–strain curve. By comparing the curves under different $f_{c}$ values, it is analyzed that as $f_{c}$ gradually increases, the stress of the entire curve after the yield point gradually decreases, among which the maximum point stress decreases from 266.03 MPa when $f_{c}$ = 0.00085 to 252.01 MPa when $f_{c}$ = 0.0034, and the fracture point stress decreases from 264.8 MPa when $f_{c}$ = 0.00085 to 245.4 MPa when $f_{c}$ = 0.0034, but the strain at the fracture point increases from 19.0% to 19.5%. It shows that with the increase in $f_{c}$, the maximum load-bearing capacity and fracture limit of the material decrease, while the elongation increases slightly. This is because increasing $f_{c}$ will make the material more susceptible to microcrack extension and coordinated void growth in the plastic stage, making the material exhibit worse bearing capacity and more brittle fracture behavior. The increase in elongation is due to the coordination effect of the voids, which delays the formation of cracks and allows the material to remain stable under slightly increased strain.

Figure 10d shows the effect of changing $f_{f}$ on the stress–strain curve. By comparing the curves under different $f_{f}$ values, it was analyzed that as $f_{f}$ gradually increased, the overall curve remained almost unchanged before fracture; the maximum point stress was about 258.03 MPa, the fracture point stress increased from 247.44 MPa at $f_{f}$ = 0.0026 to 255.01 MPa at $f_{f}$ = 0.0104 and the fracture point strain decreased from 20.2% at $f_{f}$ = 0.0026 to 19.2% at $f_{f}$ = 0.0104. It shows that as $f_{f}$ increases, the ultimate fracture strength of the material will increase, but the elongation will decrease. This is because the local stress concentration caused by void growth is more obvious. Although the damage inside the material is aggravated, the bearing capacity of the local area increases due to the increase in stress concentration, so that the material can withstand greater stress before breaking, thereby showing an increase in fracture stress. However, the plastic deformation ability of the material is weakened, causing the material to break earlier after reaching the maximum stress, resulting in a decrease in elongation.

Figure 10e shows the effect of changing $\varepsilon_{N}$ on the stress–strain curve. By comparing the curves under different $\varepsilon_{N}$ values, it is found that with the gradual increase in $\varepsilon_{N}$, both the maximum point and the fracture point change dramatically. The maximum point stress increases from 244.44 MPa when $\varepsilon_{N}$ = 0.15 to 258.03 MPa when $\varepsilon_{N}$ = 0.6, and the fracture point stress decreases from 240.29 MPa when $\varepsilon_{N}$ = 0.15 to 236.22 MPa when $\varepsilon_{N}$ = 0.6. The strain at the fracture point increases from 15.18% to 21.02%. Combined with the fact that the breaking point stress is 254.99 MPa and the strain is 19.21% when $\varepsilon_{N}$ = 0.3, it shows that enlarging or reducing the reference value will lead to a decrease in the breaking point stress, but the elongation is positively correlated with $\varepsilon_{N}$. Therefore, this parameter has the strongest sensitivity and the highest accuracy in the GTN model. This is because $\varepsilon_{N}$ represents the average strain for void nucleation. When $\varepsilon_{N}$ is large, the voids in the material require a larger strain to begin to grow significantly, which allows the material to withstand more plastic deformation before fracture. Therefore, the elongation (total strain before fracture) increases with the increase in $\varepsilon_{N}$. On the contrary, a smaller $\varepsilon_{N}$ means that the voids will grow and aggregate rapidly at lower strains, thus entering the damage stage earlier and resulting in a decrease in the elongation of the material. However, no matter whether the voids expand or shrink, grow prematurely or delay their growth, the material will suddenly break after reaching the critical point, causing the bearing capacity to drop sharply.

Figure 10f shows the effect of changing $s_{N}$ on the stress–strain curve. By comparing the curves under different $s_{N}$ values, it is analyzed that as $s_{N}$ gradually increases, the front part of the curve is almost the same, the maximum point and the fracture point have slight changes, the maximum point stress increases from 258.53 MPa when $s_{N}$ = 0.05 to 257.37 MPa when $s_{N}$ = 0.2, the fracture point stress increases from 253.36 MPa when $s_{N}$ = 0.05 to 256.19 MPa when $s_{N}$ = 0.2 and the strain at the fracture point decreases from 19.5% to 18.9%. It shows that with the increase in $s_{N}$, the maximum bearing capacity of the material increases slightly, but the stress and strain at the breaking point decrease. As $s_{N}$ increases, the dispersion of nucleation strain increases, which means that the initiation time of voids in different areas is more inconsistent. This dispersion may cause the voids in some areas to initiate later, allowing these areas to continue to bear the external load for a longer time, delaying local failure, and causing the areas where voids have not initiated to bear more stress. This stress redistribution phenomenon increases the stress value of the material at the maximum point, thereby leading to an increase in the stress at the maximum point. However, it is also easy to cause stress concentration inside the material. The premature void growth and stress concentration accelerate the fracture of the material, causing the stress and strain at the fracture point to decrease.

## 5. Conclusions

In this paper, axial and radial tensile tests on AZ31 magnesium alloy bars at different loading rates were conducted, combined with a finite element simulation of the GTN damage model, to study the mechanical behavior of the material and its influencing factors. Based on the comparison between experimental data and simulation results, the following conclusions are drawn:

(1) The mechanical properties of AZ31 magnesium alloy show obvious anisotropy in radial and axial tensile tests. Compared with the axial direction, the radial direction has a significantly lower yield strength and higher elongation, which indicates that the material’s behavior characteristics in different directions are significantly different. In addition, by conducting experiments at different tensile speeds in the axial direction, it was found that with the increase in tensile rate, the maximum stress and ductility of the material decreased, further emphasizing the influence of the loading rate on material properties. Based on this, the anisotropy of AZ31 magnesium alloys under different processing and heat treatment conditions will be further investigated alongside how these properties affect their performance in applications under complex stress states.
(2) The GTN damage model adopted in this study was simulated through the secondary development of the VUMAT user subroutine. The results showed that the model showed good accuracy and reliability in describing the tensile behavior of AZ31 magnesium alloy bars. The experimental data are highly consistent with the simulation results, which verifies the effectiveness of the GTN model and provides a solid foundation for subsequent material damage research. The validation of the GTN damage model will be subsequently extended to include a wider range of loading conditions and material states to improve the generalizability and prediction accuracy of the model.
(3) The relevant parameters of the GTN model have a certain influence on the stress–strain behavior of AZ31 magnesium alloy. Among them, the change in $\varepsilon_{N}$ has the most significant effect on the maximum stress and fracture behavior of the material. The influence of other parameters on the curve is mainly concentrated on the maximum point stress, the stress at the fracture point and the elongation. When calibrating these parameters, special attention should be paid to the accuracy of each parameter to ensure that the model can effectively reflect the performance of the material under actual loading conditions. Reasonable parameter selection will provide an important reference for the optimization and application of material properties. Afterwards, the optimization of the mechanical properties of AZ31 magnesium alloy through alloying and processing, especially the response under a high strain rate, will provide an important material selection and design basis for the application of magnesium alloy under high-speed dynamic loading.

## Figures and Tables

**Table 1:** AZ31 magnesium alloy chemical composition content (wt.%).

Elemental	Al	Si	Ca	Zn	Mn	Cu	Mg
Wt.%	3.2	0.07	0.04	1.4	0.7	0.01	Bal.

**Table 2:** Parameters of GTN damage model for AZ31 magnesium alloy.

$q_{1}$	$q_{2}$	$q_{3}$	$f_{0}$	$f_{n}$	$f_{c}$	$f_{f}$	$\varepsilon_{N}$	$s_{N}$
1.5	1	2.25	0.00045	0.013	0.0017	0.0052	0.3	0.1

## Data Availability

The datasets presented in this article are not readily available due to technical limitations. Requests to access the datasets should be directed to the corresponding author of the article, who can be reached at chuzhibing@tyust.edu.cn. Please include a brief description of your research interests and how accessing the datasets would benefit your research. The authors will consider each request on a case-by-case basis and may provide access under certain conditions such as signing a data use agreement and ensuring the confidentiality of the information.

## Abbreviations

GTN Model	Gurson–Tvergaard–Needleman Model
VUMAT	User Material Subroutine for Abaqus
Gurson Model	Gurson damage model
**ϕ**	Yield function
q $,\sigma_{eq}$	Von Mises stress
$\sigma_{m}$	Yield stress
*σ*ℎ	Hydrostatic stress
*q*1,*q*2,*q*3	Empirical parameters in the GTN model
$tr\left(\sigma \right)$	Trace of stress tensor
$\sigma_{xx},\sigma_{yy},\sigma_{zz}$	Corresponding to normal stress in x, y and z directions
f	Porosity
$f^{*}$	Current porosity, a function of porosity f
$f_{c}$	Critical pore volume fraction
$f_{u}^{*}$	Porosity value when stress is zero
$f_{f}$	Pore volume fraction at fracture
df	Increment of void fraction
$df_{grow}$	Increment of original void growth
$df_{nucl}$	Increment of new void nucleation
I	Second-order unit tensor
$f_{n}$	Volume fraction of the second phase
$\varepsilon_{p}$	Macroscopic equivalent plastic strain
$\varepsilon_{N}$	Average plastic strain of nucleation
$s_{N}$	Standard deviation, dispersion of nucleation strain
$w\left(\sigma_{ij}\right)$	Stress state function
$J_{3}$	The third invariant of the deviatoric stress tensor
